# Complete closure of an ileal perforation during enteroscopy using a novel through-the-scope twin clip

**DOI:** 10.1055/a-2844-8280

**Published:** 2026-04-20

**Authors:** Fan Zhou, Wen Li, Chunyan Peng, Ruhua Zheng, Xiwei Ding

**Affiliations:** 1 66506 Department of Gastroenterology, Nanjing Drum Tower Hospital, Aﬃliated Hospital of Medical School, Nanjing University, Nanjing, China

**Fig. 1:**
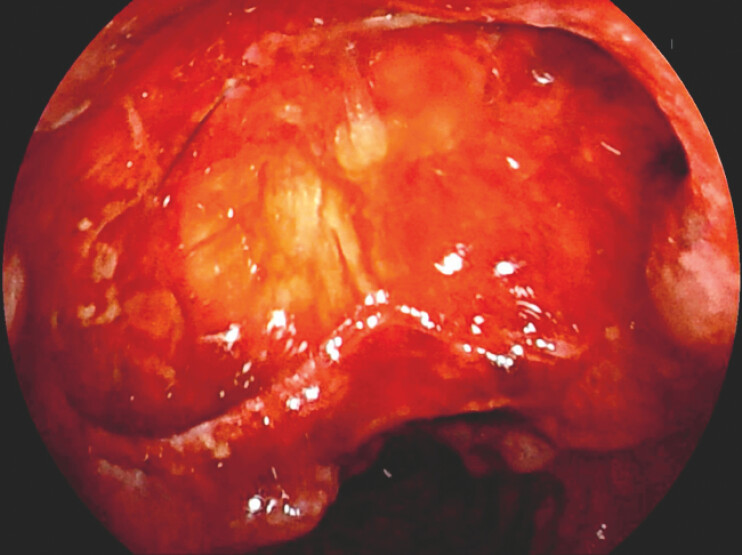
An ileal perforation occurred during transanal double-ballon enteroscopy.

**Fig. 2:**
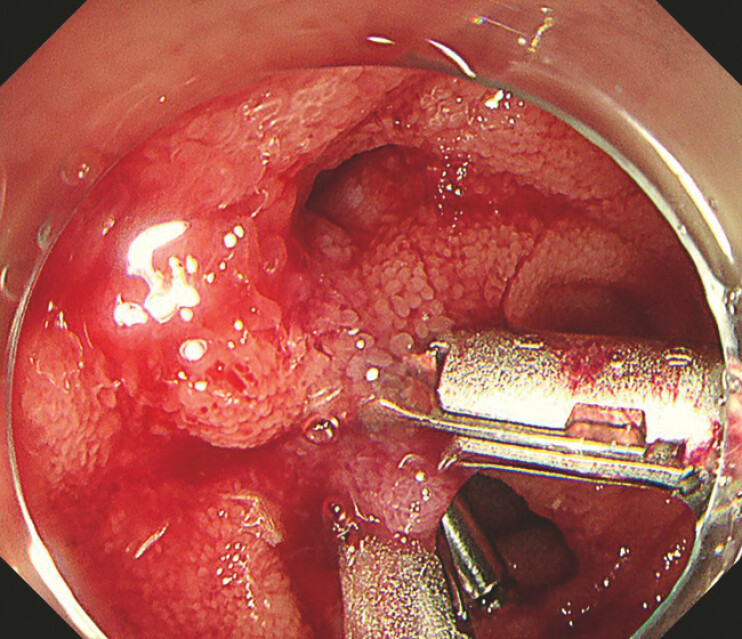
An ileal perforation sealed with a combination of a through-the-scope twin clip and conventional through-the-scope clips.

**Fig. 3:**
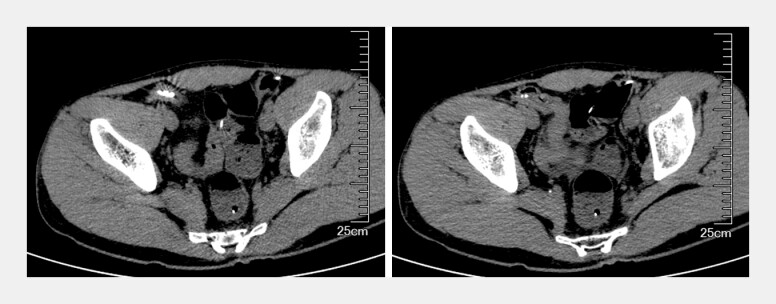
CT showed no free gas or exudation in the terminal ileum 4 days after perforation. CT, computed tomography.

A 38-year-old man with Crohn’s disease was admitted for follow-up enteroscopy after 6 months of infliximab therapy. The transanal double-balloon enteroscopy (Fujifilm, VP-7000) was discontinued after reaching the distal ileum due to severe intestinal angulation. During endoscope withdrawal, a full-thickness perforation (4 × 2 cm) was noted in the terminal ileum ( Fig. 1 ). We then switched to an Olympus endoscope and used a therapeutic colonoscopy endoscope (PCF-H290T) to reach the perforation site. First, three conventional through-the-scope clips were applied to secure the oral defect. The central part of the defect is relatively large, making it difficult to close with traditional metal clips. Subsequently, a through-the-scope twin clip (TTS-TC) was deployed to close the perforation ( Fig. 2 , Video 1 ). The TTS-TC was advanced through the biopsy channel, engaged the edges of the defect, and approximated them, thereby dividing the large opening into smaller ones. Then, 11 additional conventional through-the-scope clips were placed to achieve complete wound closure. The patient has been fasting for 4 days, undergoing gastrointestinal decompression and antibiotic therapy. A follow-up computed tomographic scan 4 days later showed no evidence of free air or exudation around the terminal ileum ( Fig. 3 ). The patient was discharged successfully on the 10 ^th^ hospital day without signs of peritonitis.

An ileal perforation during enteroscopy was sealed with a combination of a through-the-scope twin clip and conventional through-the-scope clips.Video 1

Perforation is the most common complication of double balloon enteroscopy, with a rate of 0.2%–0.4% 1 2 . The endoscopic management of ileal perforation is challenging. The TTS-TC is a novel device capable of suturing digestive tract defects 3 4 5 . In this report, we demonstrate the successful management of an ileal perforation during double-balloon enteroscopy using a combination of TTS-TC and conventional through-the-scope clips.

Endoscopy_UCTN_Code_CPL_1AI_2AD
